# Proteomic Analysis of Differentially Expressed Proteins in the Placenta of Anticardiolipin Antibody- (ACA-) Positive Pregnant Mice after Anzi Heji Treatment

**DOI:** 10.1155/2020/1967698

**Published:** 2020-12-14

**Authors:** Yazhen Xie, Qibin Lu

**Affiliations:** ^1^Nanjing University of Chinese Medicine, Nanjing, Jiangsu, China; ^2^Taicang TCM Hospital Affiliated to Nanjing University of Chinese Medicine, Taicang, Jiangsu, China; ^3^Department of Gynaecology, The Affiliated Hospital of Nanjing University of Chinese Medicine, Nanjing, Jiangsu, China

## Abstract

Anzi Heji (AZHJ) has been used to treat anticardiolipin antibody- (ACA-) positive pregnant women at risk of spontaneous abortion for many years. The aim of this study was to investigate the protective mechanism of AZHJ in a mouse model of ACA-positive pregnancy at risk of spontaneous abortion using label-free quantitative proteomics. Mice were divided into three groups: normal pregnant mice (control group), ACA-positive pregnant mice administered normal saline (model group), and ACA-positive pregnant mice administered AZHJ (AZHJ group). The model was established by injecting *β*2-glycoprotein I (GPI) into mice for 18 days. The DEPs and their functions were analyzed by label-free quantitative proteomic and bioinformatic analyses. The levels of IL-6, IL-10, ACA, and TNF-*α* in the serum and placentas of the mice were measured by enzyme-linked immunosorbent assays (ELISAs). Proteomic data were validated by western blot analysis. The abnormal serum and placental levels of IL-6, ACA, and TNF-*α* in the model group were reversed by AZHJ. There were 39 upregulated and 10 downregulated DEPs in the AZHJ group relative to the model group. Bioinformatic analysis revealed that the DEPs were mainly involved in nucleic acid binding, signal conduction, and posttranslational modification. The placental levels of T-cell immunoglobulin mucin 3 (Tim-3) and Toll-like receptor 4 (TLR4) expression and AKT phosphorylation in the three groups were consistent with the proteomic findings. Tim-3/AKT signaling is involved in maternal-fetal immune tolerance, while TLR4 is associated with inflammatory responses. Collectively, these results indicate that AZHJ may exert its protective effect in ACA-positive pregnant mice by regulating the maternal-fetal immune tolerance and inflammatory response.

## 1. Introduction

Recurrent spontaneous abortion (RSA) refers to three or more consecutive pregnancy losses [1, 2]. About 1–3% of healthy women with normal fertility and >15% of women experiencing RSA are positive for anticardiolipin antibody (ACA) [3]. ACA-positive patients have decreased *in vitro* fertilization, pregnancy, and implantation rates and an increased risk of spontaneous abortion [3, 4]. Therefore, increasing attention has been paid to the relationship between ACA and RSA.

Extracts from traditional Chinese medicine herbs or other herbs have been used to prevent abortion due to their various abilities to regulate immune responses [5–7]. Anzi Heji (AZHJ) is an approved prescription at Jiangsu Province Hospital of Traditional Chinese Medicine, China (approval no. 051226). It has been used to prevent spontaneous abortion (especially in ACA-positive cases) for many years. It has no toxic side effects [7]. The main components of AZHJ are *Cuscuta chinensis*, sangjisheng, ramie root, *Dipsacus asperata*, *Salvia miltiorrhiza*, *Atractylodes macrocephala*, *Scutellaria baicalensis*, radix Pseudostellariae, and roasted Glycyrrhizae. Our previous clinical studies revealed that AZHJ can significantly improve the clinical symptoms of ACA-positive pregnant women at risk of spontaneous abortion and improve pregnancy hormone levels [8–10]. Fetal preservation was successful in >85% of cases, and the conversion rate of ACA-positive to ACA-negative results was 90% [8–10]. However, the mechanism underlying the therapeutic effect of AZHJ remains largely unknown.

In this study, the proteomic changes in ACA-positive pregnant mice in response to AZHJ treatment were investigated using nano-high-performance liquid chromatography (HPLC)-tandem mass spectrometry (MS/MS) technology. There were 39 upregulated and 10 downregulated differentially expressed proteins (DEPs) after AZHJ treatment. This study provides a foundation for future studies investigating the therapeutic mechanisms of AZHJ.

## 2. Materials and Methods

### 2.1. AZHJ Preparation

AZHJ extraction and quality control were performed as described previously [7].

### 2.2. Animal Experiments

**Figure 1:**
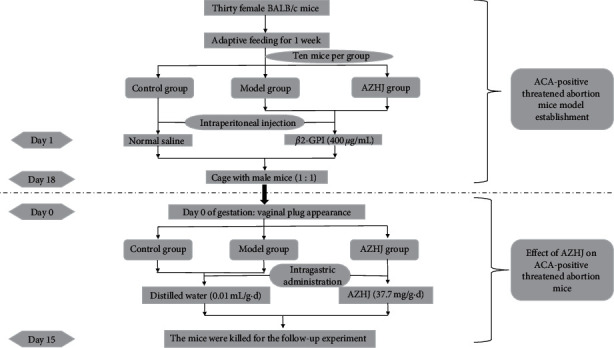
Figure of the animal experiment design and schedule.

The animal experiment design and schedule are shown in Figure 1. Eight-week-old BALB/c female (25 ± 2 g) and male (30 ± 2 g) mice were obtained from Cavens Laboratory Animal Co., Ltd (Changzhou, China). The mice were maintained in specific pathogen-free conditions at 25°C and 50–60% humidity with a 12 h/12 h light/dark cycle. The mice were fed standard chow and provided water.

The mouse model of ACA-positive pregnancy at risk of spontaneous abortion was established by injecting 400 *µ*g/mL *β*2-glycoprotein I (GPI) into 20 female BALB/c mice for 18 days, as described previously [11–13]. Another 10 female BALB/c mice were injected with normal saline for 18 days and used as the normal controls. The female mice were kept in cages with the male mice (1:1), and the day of vaginal plug appearance was considered day 0 of gestation (E0). The 20 female GPI-treated pregnant BALB/c mice were randomly divided into the AZHJ or model group. The mice in the AZHJ group were intragastrically administered 37.7 mg/g AZHJ for 15 days. The mice in the model group were intragastrically administered 0.1 mL/10 g distilled water for 15 days. The 10 normal controls were also intragastrically administered 0.1 mL/10 g distilled water for 15 days.

The pregnant mice were killed on E15. The embryo resorption rate, *R*, was calculated as *R*=Re/(Re+*F*), where Re is the number of resorbed embryos and *F* is the number of surviving embryos. The mouse experiments were carried out in accordance with guidelines approved by the Ethics Committee of Taicang Hospital of Traditional Chinese Medicine.

### 2.3. Enzyme-Linked Immunosorbent Assays (ELISAs)

The levels of ACA, TNF-*α*, IL-6, IL-2, IFN-*γ*, and IL-10 in serum samples (*n* = 6 per group) and placental tissues (*n* = 6 per group) were quantified using ELISA kits (Shanghai Joyee Biotechnics Co., Ltd., Shanghai, China) according to the manufacturer's instructions.

### 2.4. Sample Preparation and Protein Digestion

Three placental samples were obtained from each of the three groups and mixed to create a 5 *µ*l sample for each group. Radioimmunoprecipitation assay (RIPA) lysis buffer was added to the tissues, which were cut into pieces, mechanically homogenized using a tissue homogenizer three times for 3 s, and kept on ice for 15 min. After centrifugation at 12,000 g for 15 min at 4°C, the supernatant was collected and transferred to an Eppendorf tube. The proteins were redissolved in 500 mM triethylammonium bicarbonate (TEAB). The protein concentration was determined using a bicinchoninic acid (BCA) protein assay. Next, 100 *μ*g protein per group was transferred to a new tube and adjusted to a final volume of 100 *μ*L with 8 M urea. Thereafter, 11 *μ*L of 1 M dithiothreitol (DTT) was added and the sample was incubated at 37°C for 1 h and then transferred into a 10K Microcon Centrifugal Filter (Millipore, Billerica, MA, USA). To remove the urea, 100 mM TEAB was added and the samples were centrifuged, and this process was repeated twice. Next, 120 *μ*L of 55 mM iodoacetamide was added and the sample was incubated for 20 min in the dark at room temperature. The proteins were then digested with sequencing-grade modified trypsin (Promega, Madison, WI, USA) and lyophilized.

### 2.5. Liquid Chromatography with Tandem Mass Spectrometry (LC-MS/MS) Analysis

The peptides were redissolved in 30 *μ*L solvent A (A: 0.1% formic acid in water) and underwent LC-MS/MS analysis using an Orbitrap Fusion coupled to an EASY-nano-LC 1200 system (Thermo Fisher Scientific, Waltham, MA, USA). A 6 *μ*L peptide sample was loaded into a trap column (Acclaim PepMap C18 Column, 75 *μ*m × 2 cm; Thermo Fisher Scientific). They were subsequently separated using an analytical column (Acclaim PepMap C18 Column, 75 *μ*m × 50 cm) with a linear gradient from 5% B (B: 0.1% formic acid in acetonitrile) to 38% B over 120 min. The column flow rate was maintained at 300 nL/min with a column temperature of 40°C. An electrospray voltage of 2 kV was applied between the spray emitter and the inlet of the mass spectrometer.

### 2.6. Data Analysis

The MS/MS spectra were processed using PEAKS Studio v8.5 (Bioinformatics Solutions Inc., Waterloo, Canada). PEAKS DB was used to search the UniProt mouse database (v201711, 52194 entries), assuming trypsin as the digestion enzyme and setting the fragment ion mass tolerance to 0.05 Da and the parent ion tolerance to 7 ppm. Carbamidomethylation (C) was specified as the fixed modification, and oxidation (M), deamidation (NQ), and acetylation (protein N-termini) were specified as the variable modifications. The peptides were filtered based on a 1% false discovery rate and a minimum of one unique peptide per protein. Analysis of variance (ANOVA) was used for the peptide and protein abundance calculations. Normalization involved averaging the abundance of all peptides (based on median values). DEPs were defined as proteins with a fold change >1.5 and ≥2 unique peptides with significance >13 (*p* < 0.05).

### 2.7. Bioinformatic Analysis

Gene Ontology (GO) and Kyoto Encyclopedia of Genes and Genomes (KEGG) analyses were used to identify the significantly enriched biological functions and signaling pathways related to the DEPs based on the GO (geneontology.org/) and KEGG (genome.jp/kegg/) databases. To categorize the DEPs based on protein orthologs and paralogs, the Clusters of Orthologous Groups database was used (more specifically, the euKaryotic Orthologous Groups (KOGs) database; ncbi.nlm.nih.gov/COG/). Lastly, a DEP interaction network was constructed using Search Tool for the Retrieval of Interacting Genes/Proteins (STRING), as described previously (http://string-db.org/).

### 2.8. Western Blot Analysis

Total protein was extracted from placental tissues (*n* = 6 per group) using ice-cold RIPA lysis buffer containing 1% phenylmethanesulfonyl fluoride (PMSF) and a complete protease inhibitor cocktail (Beyotime Institute of Biotechnology, Haimen, China). The protein concentration was determined using a Bio-Rad Protein Assay (Bio-Rad Laboratories, Hercules, CA, USA). Next, 30 *µ*g total protein per group was separated by sodium dodecyl sulfate polyacrylamide gel electrophoresis (SDS-PAGE) and transferred to polyvinylidene difluoride (PVDF) membranes. The membranes were blocked with 5% nonfat milk for 1 h at room temperature. They were then incubated at 4°C overnight with rabbit anti-Tim-3 (1:1000; Cell Signaling Technology, Danvers, MA, USA), anti-TLR4(1:1000; Cell Signaling Technology, Danvers), anti-p-AKT(1:1000; Cell Signaling Technology), anti-AKT (1:1000; Cell Signaling Technology, Danvers), or rabbit anti-*β*-actin (1:1000; Santa Cruz Biotechnology, Santa Cruz, CA, USA), followed by adding peroxidase-labeled secondary antibody for 1 h at room temperature. The membranes were then rinsed three times (for 10 min each time) using Tris-buffered saline with Tween 20. Next, the target proteins were detected using enhanced chemiluminescence reagent (Thermo Fisher Scientific) according to the manufacturer's instructions. The relative optical density of the bands of interest was analyzed using ImageJ v1.48 (National Institutes of Health, Bethesda, MD, USA).

### 2.9. Statistical Analysis

Statistical analysis was performed using SPSS v19.0 (IBM Corp., Armonk, NY, USA). One-way ANOVA was used to analyze differences between multiple groups, and Student's *t*-test was used to analyze differences between pairs of groups. Results are expressed as the mean ± standard deviation. *p* < 0.05 was considered to indicate a statistically significant difference.

## 3. Results

### 3.1. AZHJ Reversed the Increased Embryo Loss Rate, the Increased ACA and TNF-*α* Levels, and the Decreased IL-6 Levels in *β*2-GPI-Treated Mice

**Figure 2:**
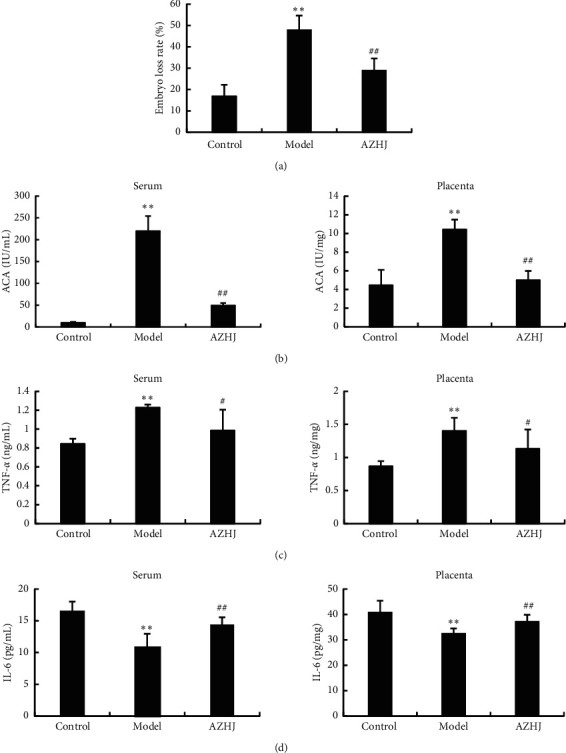
Effects of Anzi Heji (AZHJ) on embryo loss rate and the levels of ACA, TNF-*α*, and IL-6. Embryo loss rate (a) and the levels of ACA (b), TNF-*α* (c), and IL-6 (d) in serum and placentas of mice in the three groups determined by ELISAs. ^*∗∗*^*p* < 0.01 vs. control group and ^#^*p* < 0.05, ^##^*p* < 0.01 vs. model group.

The embryo loss rate of the mice in the model group was significantly higher than in the normal control group (Figure 2(a)). The model group also showed remarkable increases in the levels of ACA (Figure 2(b)) and TNF-*α* (Figure 2(c)) and decrease in the levels of IL-6 (Figure 2(d)) in serum and placentas, suggesting that the model had been established successfully. However, AZHJ reversed the *β*2-GPI-induced increases in embryo loss rate and ACA and TNF-*α* levels and the decreases in IL-6 levels. These results suggest that AZHJ attenuates the *β*2-GPI-induced responses in ACA-positive pregnant mice at risk of spontaneous abortion.

### 3.2. Identification of DEPs in Placental Tissues

Nano-HPLC-MS/MS was used to explore the protective mechanisms of AZHJ. There were 87 DEPs (35 upregulated and 52 downregulated) in the model group vs. the control group (Table 1). There were 49 DEPs (39 upregulated and 10 downregulated) in the AZHJ group vs. the model group (Table 1).

### 3.3. Bioinformatic Analysis of DEPs

**Figure 3:**
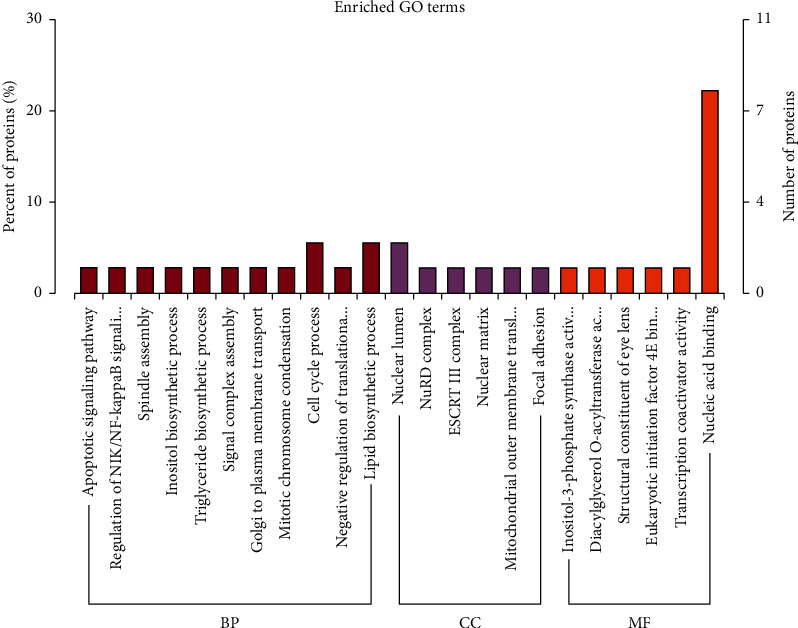
GO annotation of the DEPs (AZHJ vs. model group) in the biological process (BP), cellular component (CC), and molecular function (MF) categories.

The DEPs (AZHJ vs. model group) were analyzed with regard to GO cellular components (CC), molecular functions (MF), and biological processes (BP) using InterProScan. Regarding the BP terms, the majority of the DEPs were associated with cell cycle process and lipid biosynthetic process (Figure 3). Regarding the CC terms, the DEPs were most commonly associated with nuclear lumen (Figure 3). Lastly, regarding the MF terms, the DEPs were most commonly associated with nucleic acid binding (Figure 3).

**Figure 4:**
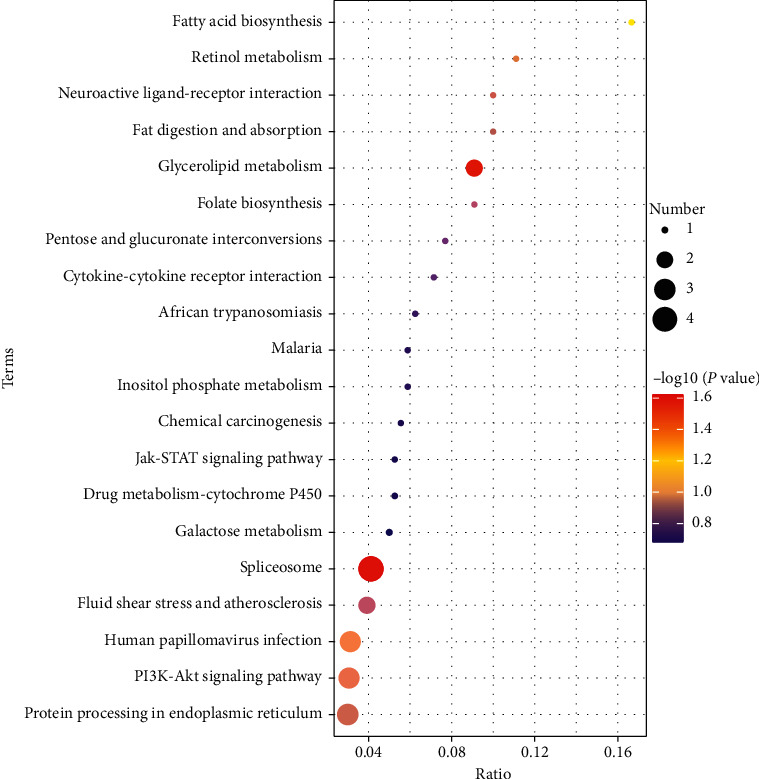
Distribution of enriched KEGG pathways. Circles represent numbers of DEPs, which are colored from blue (smaller *p* values) to red (larger *p* values).

The KEGG pathway analysis showed that the DEPs implicated in the protective effects of AZHJ were mainly involved in signaling pathways related to spliceosomes, human papillomavirus infection, the phosphoinositide 3-kinase (PI3K)-AKT signaling pathway, and protein processing in the endoplasmic reticulum (Figure 4).

**Figure 5:**
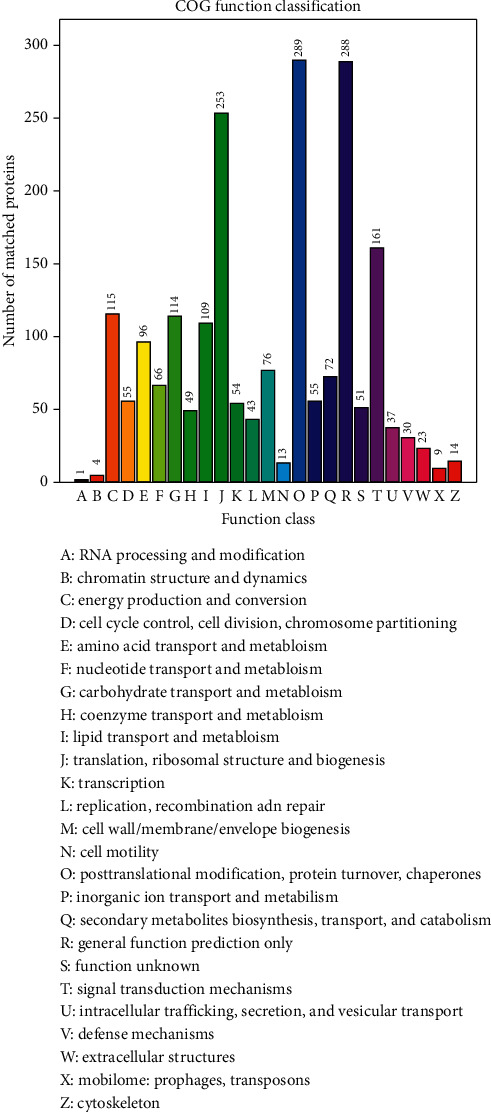
Cluster of Orthologous Groups (COG) analysis of DEPs (AZHJ vs. model group). Different colors represent different functions. The ordinate value represents the number of DEPs enriched in each function.

The COG analysis showed that the DEPs were associated with posttranslational modification, protein turnover, chaperones, translation, ribosomal structure, and biogenesis (Figure 5).

**Figure 6:**
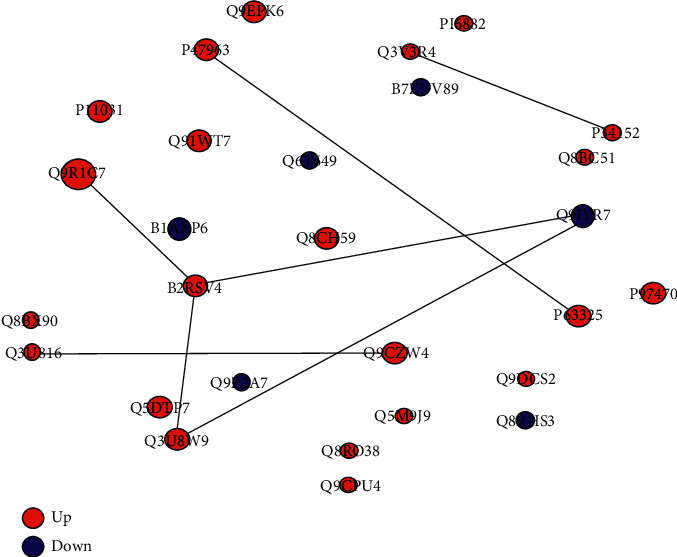
DEP interaction network. DEP interactions were determined by searching the Search Tool for the Retrieval of Interacting Genes/Proteins (STRING) database v9.0 with a confidence cutoff of 0.6. In the network, the nodes represent DEPs and the connecting lines represent interactions.

To better understand the protective mechanisms of AZHJ, DEP interaction networks were constructed using STRING. As shown in Figure 6, most of the DEPs in the interaction network exhibited direct or indirect links.

### 3.4. Validation of DEPs by Western Blotting

**Figure 7:**
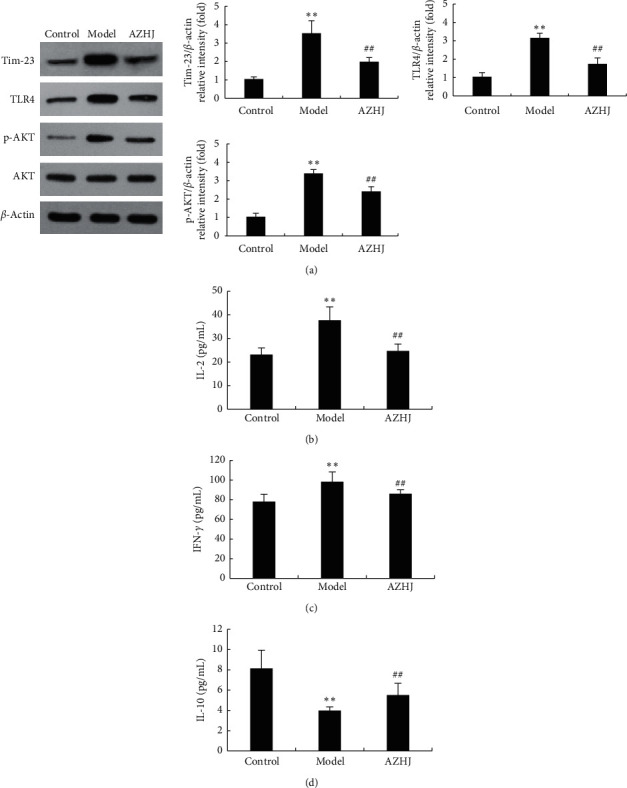
Western blot analysis confirmed DEPs identified by the quantitative proteomic analysis. (a) Tim-3, TLR4, *p*-AKT, and AKT expression in placentas of mice in the three groups analyzed by western blot analysis. IL-2 (b), IFN-*γ* (c), and IL-10 (d) levels in serum of mice in the three groups determined by ELISAs. ^*∗∗*^*p* < 0.01 vs. control group, ^#^*p* < 0.05, and ^##^*p* < 0.01 vs. model group.

The levels of Tim-3 and Toll-like receptor 4 (TLR4) expression and AKT phosphorylation were significantly increased in the model group compared to the control group (*p* < 0.05, Figure 7(a)). However, the levels were significantly reduced by AZHJ. These findings were consistent with those derived from the proteomic analysis. In addition, serum IL-2 and IFN-*γ* were increased and serum IL-10 was decreased in the model group compared to the control group (*p* < 0.05, Figures 7(b)–7(d)), and these changes were reversed in the AZHJ group (*p* < 0.05, Figures 7(b)–7(d)).

## 4. Discussion

This study is the first to use a quantitative proteomic analysis to identify the DEPs in placental tissues from ACA-positive pregnant mice after AZHJ treatment. After AZHJ treatment, the serum and placental levels of ACA and TNF-*α* were decreased, while the serum and placenta levels of IL-6 were increased. These findings suggest that AZHJ exhibited a significant therapeutic effect in ACA-positive pregnant mice. ACA induces abortion via promoting thrombosis, damaging trophoblasts, and activating inflammation and the complement response by targeting *β*2-GPI [14, 15]. TNF-*α* plays a critical role in the physiology and pathology of pregnancies by regulating the immune balance at the maternal-fetal interface [16, 17]. Excessive TNF-*α* causes thrombosis and TNF-*α* can activate immunocompetent cells in the decidual stroma, induce immune-mediated destruction and immune rejection, hinder the maintenance of pregnancy and embryo implantation, disrupt the homeostasis of the maternal uterine environment, and cause spontaneous abortion [16, 18, 19]. IL-6, which is produced by extravillous trophoblasts and cytotrophoblasts, plays an important role in the normal placental development and pregnancy success by regulating placental cell migration and invasion and trophoblast differentiation and proliferation [20, 21]. However, the role of IL-6 in ACA-positive spontaneous abortion is controversial, as Krause et al. [22] showed that the serum IL-6 was increased in ACA-positive pregnant mice, while Karakantza et al. [23] reported the opposite result. Our previous studies showed that serum IL-6 in ACA-positive pregnant mice at risk of spontaneous abortion was significantly increased, but there were no changes in the placental or decidual IL-6 levels [11–13]. Thus, we speculate that IL-6, as a proinflammatory factor, plays a key role in the inflammatory reaction, which may be related to ACA production and ACA-induced intravascular lesions, inflammatory responses, and thrombosis.

Our label-free quantitative proteomic analysis identified 49 DEPs in the placentas of ACA-positive pregnant mice after AZHJ treatment. GO and KEGG analyses further revealed that the significantly enriched biological functions and signaling pathways included nucleic acid binding, the PI3K-AKT signaling pathway, and protein processing in the endoplasmic reticulum. These GO terms and KEGG pathways may shed light on the mechanisms underlying the therapeutic effect of AZHJ. According to the COG analysis, the DEPs were predominantly enriched in posttranslational modification, protein turnover, chaperones, and translation, ribosomal structure, and biogenesis.

Th1 and Th2 cell-mediated immune regulation is a major mechanism of maternal peripheral immune tolerance [16, 24]. There is a significant Th2 bias in normal pregnancy, while spontaneous abortion is often associated with a Th1 bias [24]. Th1 cytokines (including IL-2, IFN-*γ*, and TNF-*β*) are involved in inflammatory response and immune response [16, 24]. Th2 cells produce anti-inflammatory IL-4, IL-5, IL-6, and IL-10, which contribute to the success of pregnancy [16, 24]. IL-6 contributes to normal embryo implantation in early pregnancy [16, 24]. As an immunosuppressive factor, IL-10 plays an important role in maintaining immune tolerance [16, 24]. In addition, IL-10 can downregulate Th1 cytokines in macrophages and inhibit NF-*κB*, thus playing an immunomodulatory role [16, 24].

Tim-3 is a Tim family receptor protein that is a type I transmembrane protein [25]. It is expressed in a variety of cells, including Th1, Th17, NK, and NKT cells, Tregs, dendritic cells, monocytes, macrophages, and decidual stromal cells [26]. It plays an important regulatory role in the occurrence and development of female reproductive system diseases and is closely related to the occurrence of spontaneous abortion [26]. Blocking Tim-3 *in vitro* decreases IL-4 and IL-10 production by CD_8_^+^ T cells, increases IFN-*γ* production, disrupts the Th1/Th2 balance, and affects the maternal-fetal tolerance [27]. Galectin-9 (Gal-9), which is related to the activation of T cells, is a ligand of Tim-3 [26]. High Tim-3 expression in the peripheral blood and decidual tissues of patients with RSA has been reported to be related to the occurrence and development of RSA [28, 29]. Other studies reported that soluble Tim-3 and Gal-9 are increased in the serum of patients with unexplained RSA, which indicated that the soluble costimulatory molecule Tim-3 may regulate the differentiation of Th1 and Th2 cells in patients with unexplained RSA, with the Th1/Th2 balance being biased toward Th1 [30–32]. The combination of Tim-3 and Gal-9 may weaken the Tim-3/Gal-9 signaling pathway and transmit inhibitory signals, thus participating in the occurrence and development of unexplained RSA. In the present study, the placental level of Tim-3 protein was significantly increased in the ACA-positive pregnant mice and significantly decreased after AZHJ treatment. The differences in the serum levels of Th1/Th2-type cytokines (such as IL-2, IFN-*γ*, and IL-10) between the model and AZHJ groups might be at least partly attributable to the altered Tim-3 expression.

The PI3K-AKT signaling pathway plays an important role in the occurrence and development of RSA (including unexplained RSA) [33, 34]. Inhibition of this pathway promotes trophoblast apoptosis and suppresses trophoblast proliferation and migration [33, 34]. This pathway also controls the differentiation of Treg and Th17 cells [35]. We found that the phosphorylation level of AKT in the placentas of ACA-positive pregnant mice was increased significantly, while it was decreased by AZHJ. PI3K-AKT signaling is an important downstream target of Tim-3 [36]. Furthermore, Tim-3/Gal-9 signaling in peripheral NK cells promotes maternal-fetal tolerance by regulating the PI3K-AKT signaling pathway [28]. Thus, we speculated that the reduced phosphorylation level of AKT may be due to the downregulation of Tim-3 expression by AZHJ treatment.

TLRs play important roles in reproductive processes such as ovulation, spermatogenesis, sperm capacitation, fertilization, and pregnancy [37]. A recent study showed that decreased TLR4 expression in response to ligand treatment of spermatozoa is associated with unexplained RSA [38]. However, a study by Li et al. [39] revealed that increased TLR4 expression in decidual and chorionic tissues was closely related to the occurrence of RSA. In the present study, increased TLR4 expression was observed in the placentas of ACA-positive pregnant mice. To the best of our knowledge, the present study is the first to evaluate TLR4 expression in ACA-positive pregnant mice at risk of spontaneous abortion. Our results revealed that TLR4 expression was significantly decreased by AZHJ, suggesting that it may be a critical downstream target of AZHJ. Tim-3 is involved in regulating TLR4 signaling, and whether TLR4 was regulated by Tim-3 after AZHJ treatment needs to be investigated in the future.

## 5. Conclusion

This study provides evidence that AZHJ exerts a protective effect against spontaneous abortion in ACA-positive pregnant mice. Our data suggest that the mechanisms underlying these effects include inhibiting Tim-3/AKT signaling and downregulating TLR4. Our data provide guidance and a useful foundation for investigating new treatments for ACA-positive spontaneous abortion.

## Figures and Tables

**Table 1:** Number of differentially expressed proteins (DEPs) in placenta from control, model, and AZHJ groups.

Groups	Number of DEPs	Number of upregulated proteins	Number of downregulated proteins
Control group vs. model group	87	35	52
Model group vs. AZHJ group	49	39	10
Control group vs. AZHJ group	103	72	31

## Data Availability

All of the data reported in this article are available from the corresponding author upon reasonable request.
